# A Recent Cough

**Published:** 1880-01

**Authors:** 


A Recent Cough will almost always yield to the following treatment within two or three days : Mix in a bottle 4 ounces of glycerine, 2 ounces of alcohol, 2 ounces of water, 2 grains of morphine. Shake well. Dose for an adult, 1 to 2 teaspoonfuls every 2 or 3 hours. Half this quantity to children from 10 to 15 years. It is not safe to give it to infants or children under 10 years of age.

